# Latent Membrane Protein 1 as a molecular adjuvant for single-cycle lentiviral vaccines

**DOI:** 10.1186/1742-4690-8-39

**Published:** 2011-05-18

**Authors:** Sachin Gupta, James M Termini, Liguo Niu, Saravana K Kanagavelu, Andrew R Rahmberg, Richard S Kornbluth, David T Evans, Geoffrey W Stone

**Affiliations:** 1 Department of Microbiology & Immunology, University of Miami Miller School of Medicine, Miami, FL, USA; 2 Department of Microbiology and Molecular Genetics, Harvard Medical School, New England Primate Research Center, Southborough, MA, USA; 3 Multimeric Biotherapeutics, Inc., La Jolla, CA, USA; 4 University of North Carolina School of Medicine, 3302 Michael Hooker Research Building, Chapel Hill, NC, 27599, USA

## Abstract

**Background:**

Molecular adjuvants are a promising method to enhance virus-specific immune responses and protect against HIV-1 infection. Immune activation by ligands for receptors such as CD40 can induce dendritic cell activation and maturation. Here we explore the incorporation of two CD40 mimics, Epstein Barr Virus gene LMP1 or an LMP1-CD40 chimera, into a strain of SIV that was engineered to be limited to a single cycle of infection.

**Results:**

Full length LMP1 or the chimeric protein LMP1-CD40 was cloned into the *nef*-locus of single-cycle SIV. Human and Macaque monocyte derived macrophages and DC were infected with these viruses. Infected cells were analyzed for activation surface markers by flow cytometry. Cells were also analyzed for secretion of pro-inflammatory cytokines IL-1β, IL-6, IL-8, IL-12p70 and TNF by cytometric bead array.

**Conclusions:**

Overall, single-cycle SIV expressing LMP1 and LMP1-CD40 produced a broad and potent T_H_1-biased immune response in human as well as rhesus macaque macrophages and DC when compared with control virus. Single-cycle SIV-LMP1 also enhanced antigen presentation by lentiviral vector vaccines, suggesting that LMP1-mediated immune activation may enhance lentiviral vector vaccines against HIV-1.

## Background

To develop an effective lentiviral vector vaccine against HIV-1 infection it may be necessary to focus on enhancing the activation of dendritic cells, and other professional antigen presenting cells, in order to maximize the stimulation of virus-specific immune responses. One of the critical events in the induction of immune response is the maturation of DCs and macrophages [1]. Maturing DCs and macrophages undergo a rapid burst of cytokine synthesis and expression of costimulatory molecules. Dendritic cells then migrate to the T-cell areas of draining secondary lymphoid organs to prime naïve T cells and initiate an adaptive immune response [2]. IL-12p70 is secreted by activated macrophages and DC and stimulates IFN-γ secretion by T lymphocytes and NK cells [1,3,4]. To improve the efficacy of vaccines, we decided to focus on developing single-cycle SIV vaccines incorporating inducers of antigen presenting cell maturation and cytokine secretion, specifically looking at CD40 stimulation and the role of the viral protein LMP1.

LMP1 is an integral membrane protein of Epstein Barr Virus (EBV) with a molecular weight of approximately 63 kDa, consisting of three domains. LMP1 expression induces many of the changes associated with EBV infection and activation of primary B cells, including cell clumping; increased cell surface expression of CD23, CD39, CD40, CD44; decreased expression of CD10; and increased expression of the cell adhesion molecules CD11a (LFA1), CD54 (ICAM1), and CD58 (LFA3) [5-8]. At least four signaling pathways, namely nuclear factor κB (NF-κB), c-Jun N-terminal kinase (JNK)-AP-1, p38/MAPK (mitogen activated protein kinase), and Janus kinase (JAK)-STAT (signal transducers and activators of transcription), are implicated in the function of LMP1 [9-12]. Within the C-terminus of LMP1 there are at least two activating regions referred to as CTAR1 and CTAR2 (C-terminal activating region). CTAR1 is located proximal to the membrane (amino acids 186-231) and is essential for EBV mediated transformation of primary B cells. CTAR2 (amino acids 351-386) is located at the extreme C-terminus of LMP1 and is required for long term growth of EBV positive primary B cells [13,14]. Both CTAR1 and CTAR2 can activate NF-κB independently [9]. Aggregation of LMP1 within the plasma membrane is a crucial prerequisite for signaling. LMP1 aggregation appears to be an intrinsic property of the transmembrane domain [15]. This signaling is similar to signaling by the tumor necrosis factor receptor (TNFR) CD40 [16]. The main difference between LMP1 and the TNFR family is that LMP1 functions as a constitutively activated receptor and, therefore, does not rely on the binding of an extracellular ligand for costimulation [17]. Experiments have also evaluated the chimeric molecule LMP1-CD40, consisting of the LMP1 transmembrane domain and the CD40 cytoplasmic tail. These experiments suggest that the LMP1-CD40 chimera is also constitutively active in vitro [18].

In this study, we took advantage of the immunostimulatory characteristics of LMP1 and LMP1-CD40 by incorporating these genes into the genome of pseudotyped single-cycle SIV viral particles. These genes are expected to enhance the immunogenicity of the virus, thereby stimulating antigen presentation by infected APC. We evaluated the immunogenicity of SIV-LMP1 and SIV-LMP1-CD40 in vitro using human as well as macaque monocyte-derived DC and macrophages. Our data suggest that LMP1 and LMP1-CD40 significantly enhance the ability of SIV to activate DCs and macrophages. SIV-LMP1 also enhances the priming of naive Gag-specific T cells in vitro. These results are encouraging for the clinical evaluation of LMP1 and LMP1 chimeric constructs as a novel class of adjuvant for HIV vaccines and other immunotherapy strategies.

## Results

### Preparation of LMP1 and LMP1-CD40

**Figure 1:**
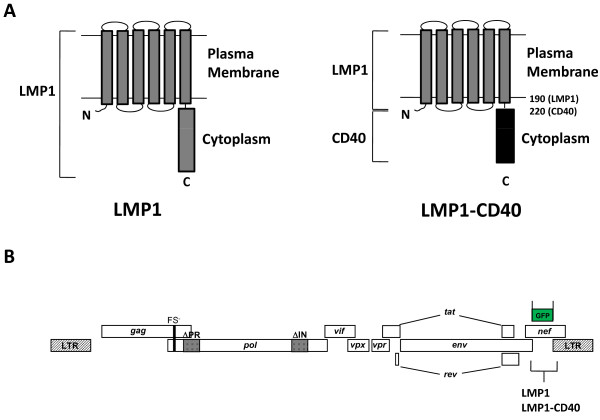
**Schematic of LMP1 constructs and single cycle SIV genome**. (A) Representation of LMP1 functional domains and the LMP1-CD40 chimeric protein. The LMP1 N-terminal transmembrane region enables the formation of clusters in the plasma membrane. This clustering is essential for LMP1 activity. In the LMP1-CD40 fusion protein, the cell signaling C-terminal region of LMP1 has been replaced and amino acid 190 of LMP1 is linked to the intracellular domain of the CD40 receptor, beginning at amino acid 220 the CD40 protein. (B) Schematic of scSIV viral genome. The parent vector, expressing GFP from the Nef promoter, was cloned by overlap PCR and inserted into the SIV_mac_239 FS-ΔPR-ΔINEGFP vector using unique XbaI and SacII sites. To create immunostimulatory forms of scSIV, LMP1 or LMP1-CD40 were inserted in place of the GFP gene as shown.

Both LMP1 and LMP1-CD40 chimera genes were constructed from PCR fragments, using Raji B cell line cDNA and human CD40 cDNA as PCR templates. The resulting proteins are depicted in Figure 1A. The LMP1 N-terminal residues form a domain with six transmembrane regions that self-associate in the plane of the membrane, clustering the cytoplasmic tails of the protein. The cytoplasmic tail, either from LMP1 or CD40, contains signaling domains that recruit adapter molecules such as TRAFs to initiate downstream signaling events. Receptor-ligand interaction is not required to induce clustering, and as a result both LMP1 and LMP1-CD40 are constitutively active [18].

### Generation of pseudotyped single-cycle SIV expressing LMP1 or LMP1-CD40

**Figure 2:**
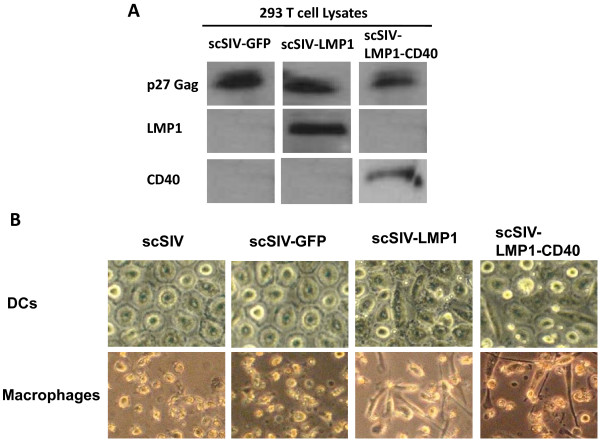
**SIV virus expressing LMP1 or LMP1-CD40 induces morphological changes in DCs and macrophages**. (A) Western blot of 293T cell lysates transfected with SIV expressing LMP1 or LMP1-CD40. Virus expressing GFP served as a negative control. Gag p27 was present in all lysates. LMP1 and CD40 intracellular domains were present only in cells transfected with LMP1 or LMP1-CD40 viral constructs respectively. Blots were stained with anti-Gag (upper panels), anti-LMP1 intracellular domain (middle panels), or anti-CD40 intracellular domain (lower panels). (B) Representative images of human monocyte derived dendritic cells (DCs) or macrophages infected with the various SIV viruses. DCs or macrophages were infected with the parent single cycle virus SIV_mac_239 FS-ΔPR-ΔIN (expressing Nef) (10) or a Nef-deleted scSIV expressing GFP, LMP1, or LMP1-CD40. Only LMP1 or LMP1-CD40 expressing viruses induced elongation of human DCs or macrophages, suggesting the activation and maturation of cells in the culture.

The single-cycle SIV viral construct scSIV_mac_239FS-ΔPRΔINEGFP [19] was used as a template to generate single-cycle SIV virus expressing either LMP1 or LMP1-CD40 (Figure 1B). After confirming recombinant clones by sequencing we performed Western blot analysis for Gag, LMP1, and CD40 following transfection of 293T cell lysates with SIV viral constructs. Gag (p27) was present in all 293T lysates, whereas LMP1 and CD40 proteins were present only for LMP1 and LMP1-CD40 adjuvanted viruses, respectively (Figure 2A). Theoretical molecular weights of LMP1 (42 kDa) and LMP1-CD40 (28 kDa), were consistent with Western blot values (40 kDa and 30 kDa respectively).

### Transduction of human DCS and macrophages with SIV encoding LMP1 and LMP1-CD40 results in enhanced activation and maturation

**Figure 3:**
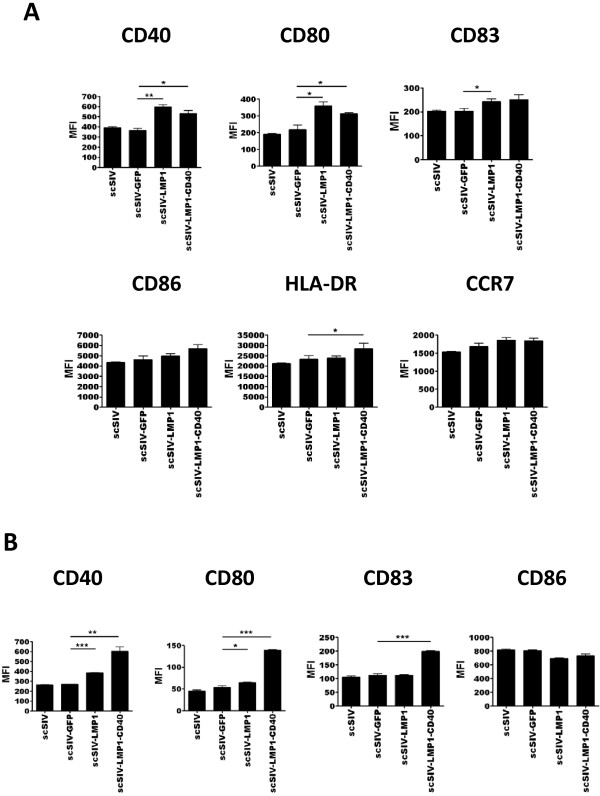
**Transduction of Human DCs or macrophages with scSIV expressing LMP1 or LMP1-CD40 results in increased levels of maturation and activation markers**. The expression levels of surface markers from three independent experiments are presented as mean fluorescence intensity (MFI). (A) The expression of surface markers on SIV infected DCs was examined by flow cytometry 4 days after transduction. Transduction with scSIV-LMP1 resulted in dendritic cell activation and maturation as measured by significantly increased levels of CD40, CD80 and CD83 expression, while scSIV-LMP1-CD40 resulted in significant increased levels of CD40, CD80 and HLA-DR expression when compared to scSIV-GFP-transduced cells. (B) The expression level of surface markers on scSIV virus-transduced macrophages was examined 4 days after transduction by flow cytometry from a representative donor. Transduction with scSIV-LMP1 resulted in increased levels of CD40 and CD80 expression, while scSIV-LMP1-CD40 resulted in increased levels of CD40, CD80 and CD83 expression compared to scSIV-GFP-transduced macrophages. As the positive control for the maturation and activation, MIMIC cytokine cocktail for DCs and LPS was used for macrophages. Data were analyzed with the unpaired t test: *, p < 0.05; **, p < 0.01; ***, p < 0.001 compared with the scSIV-GFP infected group.

Viruses expressing LMP1, LMP1-CD40, or control GFP were tested for their ability to activate human DCS and macrophages. Initially we determined the optimal infectious dose as MOI of 0.05 and optimal time for analysis as 4 days post infection (Additional file 1, Figure S1). Under these conditions, scSIV expressing LMP1 or LMP1-CD40 induced morphological changes in DCs and macrophages, including clumping and elongation of cells within the culture (Figure 2B). Similar morphological responses were also observed after treatment with LPS, suggesting that LMP1 and LMP1-CD40 are inducing activation of cells within the infected cultures (data not shown). Next we tested the expression levels of various maturation and activation surface markers on virus-transduced macrophages and DCs by flow cytometry. Cells were again evaluated 4 days after infection with scSIV viruses. Transduction with scSIV-LMP1 resulted in dendritic cell activation and maturation as measured by significantly increased levels of CD40, CD80 and CD83 expression, while scSIV-LMP1-CD40 resulted in significant increased levels of CD40, CD80 and HLA-DR expression when compared to scSIV-GFP-transduced cells. (Figure 3A). These results suggest that the activation signal provided by LMP1 and LMP1-CD40 is strong enough to initiate both activation and maturation of DCs. Similarly, there was a significant increase in the expression of maturation markers CD40 and CD80 on scSIV-LMP1 transduced macrophages, whereas scSIV-LMP1-CD40 resulted in an increase in the expression levels of CD40, CD80 and CD83 (Figure 3B).

### scSIV expressing LMP1 or LMP1-CD40 result in increased secretion of inflammatory cytokines and β-chemokines from human and macaque DCs and macrophages

**Figure 4:**
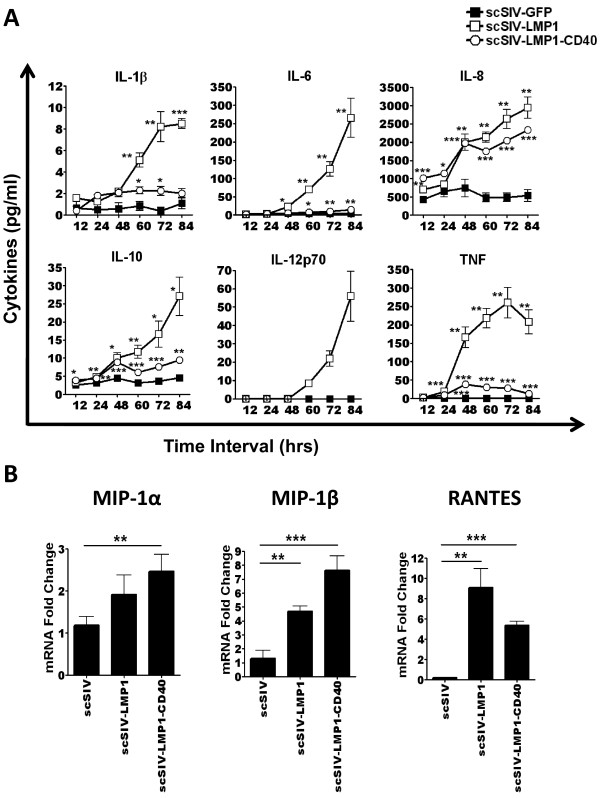
**scSIV expressing LMP1 or LMP1-CD40 induces increased secretion of inflammatory cytokines and β-chemokines**. Human inflammatory cytokine quantitation was performed by cytometric bead array (CBA). Cytokine concentrations from three independent experiments are presented. Data were analyzed with the unpaired t test: *, p < 0.05; **, p < 0.01; ***, p < 0.001 compared with the scSIV-GFP infected group. MIP-1α, MIP-1β, RANTES mRNA expression was analyzed by real-time RT-PCR assay. (A) DCs were infected with the various SIV viruses at MOI of 0.05 and supernatants were collected at various time intervals. Virus expressing LMP1 resulted in a significant increase in IL-1β, IL-6, IL-8, IL-10, IL-12p70 and TNF, while LMP1-CD40 resulted in an increase in IL-1β, IL-6, IL-8, IL-10 and TNF at various time points post infection. No measurable amounts of IL-12p70 were detected in GFP and LMP1-CD40 groups. (B) Macrophages were infected with different scSIV viruses for 4 days. Total cellular RNA was isolated, reverse transcribed to cDNA and MIP-1α, MIP-1β, RANTES mRNA expression was analyzed by real-time PCR assay. Virus expressing LMP1 resulted in significant increase in MIP-1β and RANTES mRNA expression, whereas LMP1-CD40 resulted in significant increase in MIP-1α, MIP-1β and RANTES mRNA expression. Expression of GAPDH was used for normalization of samples.

We next examined the secretion of various human inflammatory cytokines by virus-infected DCs or macrophages. Inflammatory cytokine assays were performed by cytometric bead array (CBA). DCs were infected with different single-cycle SIV viruses at MOI of 0.05 and supernatants were collected at various time intervals. scSIV-LMP1 infection resulted in a significant increase in IL-1β, IL-6, IL-8, IL-10, IL-12p70 and TNF, while scSIV-LMP1-CD40 infection resulted in increase in IL-1β, IL-6, IL-8, IL-10 and TNF at various time points (Figure 4A). Moreover, we could not detect measurable amounts of IL-12p70 in scSIV-GFP or scSIV-LMP1-CD40 infected DCs. Additional file 2, Table S1 summarizes the concentration and p-values for cytokines secretion from infected human and macaque DCs and macrophages. Values for scSIV-LMP1 or scSIV-LMP1-CD40 were compared to scSIV-GFP. We observed significantly higher secretion of inflammatory cytokines from macaque DCs and macrophages upon infection with LMP1 and LMP1-CD40 adjuvanted scSIV viruses compared to control virus. In all assays LPS was used as a positive control and induced high levels of IL-8, IL-6, and TNF from both dendritic cells and macrophages (data not shown). These results confirm that LMP1 and LMP1-CD40 are able to activate DCs and macrophages in vitro both in humans and non-human primates. These data show that incorporating LMP1 and LMP1-CD40 into SIV enhances its ability to activate DCs and macrophages. We also evaluated β-chemokine RNA expression by real time RT-PCR of macrophages 4 days following infection. Total cellular RNA was isolated, reverse transcribed to cDNA and MIP-1α, MIP-1β, and RANTES mRNA expression was analyzed by real time PCR assay. When macrophages were infected with recombinant scSIV viruses, LMP1 resulted in a significant increase in MIP-1β and RANTES mRNA expression, whereas LMP1-CD40 resulted in significant increase in MIP-1α, MIP-1β and RANTES mRNA expression (Figure 4B). Taken together, these results suggest that expression of both pro-inflammatory cytokines and β-chemokines is enhanced by single cycle SIV expressing LMP1 or LMP1-CD40.

### SIV-LMP1 infected DCs can enhance antigen-specific immune responses from autologous T cells

**Figure 5:**
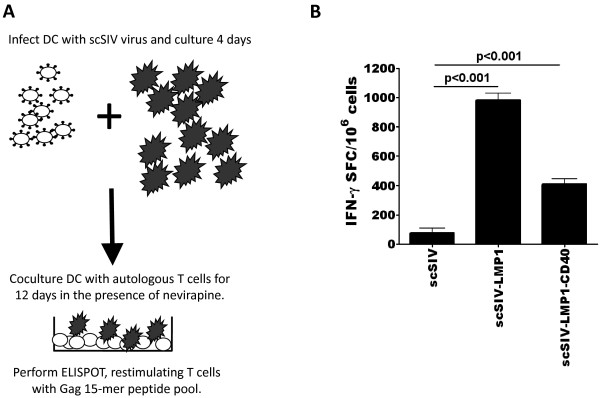
**LMP1 induces enhanced T cell responses in a Gag peptide-specific IFN-γ ELISPOT assay**. (A) Schematic of the experimental protocol. DCs from an HIV seronegative donor were transduced with scSIV viruses for 4 days, washed, and then incubated with autologous T cells for 12-days in the presence of nevirapine and IL-2 (5 U/ml) starting on day 3 of the coculture. After 12 days, cultures were restimulated with a consensus SIV_mac_239 15-mer Gag peptide pool and IFN-γ ELISPOT analysis was performed 24 hours later. (B) DCs infected with parent scSIV were unable to stimulate anti-SIV T cell responses, while the nef-deleted virus scSIV-GFP induced a modest T cell response. DC infected with scSIV-LMP1 and LMP1-CD40 significantly enhanced anti-Gag T cell responses (p < 0.001). Results are representative of three independent experiments using three different donor blood samples.

IL-12p70 is an important regulator of IFN-γ secretion by T cells [22]. We therefore investigated whether the conditions that induce IL-12p70 production by scSIV-LMP1 transduced DCs can also increase IFN-γ secretion by autologous T cells following DC stimulation in a 12-day DC:T cell co-culture assay. DCs were transduced with scSIV-LMP1, scSIV-LMP1-CD40 or scSIV for 4 days, washed, and then cultured with autologous T cells for 12 days in the presence of nevirapine and 5 u/ml of IL-2 (Figure 5A). T cells were then restimulated with an SIV Gag 15-mer overlapping peptide pool (NIH AIDS reagent program). IFN-γ secreting cells were identified by ELISPOT analysis. The scSIV-LMP1 and scSIV-LMP1-CD40 infected DC induced an increased IFN-γ T cell response as compared to the scSIV control (Figure 5B).

## Discussion

In the present study, we investigated methods to develop safe and efficacious SIV vaccines by incorporating adjuvant genes LMP1 and LMP1-CD40 into the genome of single cycle SIV. These and similar vaccination strategies are based on the activation of DCs and macrophages via CD40 signaling, resulting in an inflammatory response that is able to enhance antigen-specific T cell responses in the vaccine. This CD40 signaling may be especially critical in eliciting CTL responses in conditions such as AIDS during which the number or activity of CD4+ T cells is limited. The incorporation of LMP1 and LMP1-CD40 into scSIV viral particles resulted in enhanced immunogenicity compared to parent scSIV as evidenced by the induction of T_H_1 cytokines and both DC and macrophage maturation. The scSIV viral genome was efficiently recombined with LMP1, LMP1-CD40 and these proteins were expressed as confirmed by western blot. As an indication of the potency of the LMP1 adjuvants, scSIV viruses expressing LMP1 and LMP1-CD40 induced morphological changes in DCs and macrophages, including clumping and elongation suggestive of activation of these cells.

The immunogenicity of scSIV incorporating LMP1 and LMP1-CD40 was next evaluated in vitro by measuring the expression levels of cell surface markers transduced DCs and macrophages. The expression levels of maturation markers CD40, CD80, CD83, CCR7 and HLA-DR were higher in LMP1 and LMP1-CD40 adjuvanted scSIV transduced DCs as compared to the GFP control group. The expression of CD40, CD80, CCR7, and HLA-DR were similar to positive control cells matured with cytokines, however the expression level of CD83 on LMP1 and LMP1-CD40 virus transduced DC was not as high as that on cytokine cocktail-maturated DCs (data not shown), suggesting that transduction with LMP1 and LMP1-CD40 did not induce complete maturation of the DCs and macrophages in vitro. This was not unexpected, since we intentionally excluded other activating stimuli in the culture medium to increase the sensitivity of the assay. This could be explained by the fact that the MOI used in the infection experiments was very low (0.05). This low MOI led to a low level of transfection, normally 10-20% of cells exposed to SIV constructs. These results suggest that the activation signal provided by LMP1 and LMP1-CD40 is potent enough to initiate activation but not enough to induce full maturation. There was also a modest increase in the expression of maturation markers such as CD40 and CD83 in LMP1 adjuvanted scSIV transduced macrophages, whereas LMP1-CD40 adjuvanted scSIV resulted in a marked increase in the expression levels of CD40, CD80 and CD83 on infected macrophages. These differences suggest a positive feedback whereby CD40 signaling from LMP1-CD40 enhances the expression of CD40 protein on the cell surface.

Overall, T_H_1 cytokine secretion was dramatically enhanced by SIV encoding LMP1, with increased cytokine secretion observed within 12-24 hours post-infection. This rapid cytokine induction included a modest level of IL-12p70, suggesting that SIV-LMP1 infected DCs could potentially enhance SIV-specific T cell response in vivo. This is balanced with a modest secretion of IL-10 following scSIV-LMP1 infection of DC. Much greater secretion levels were observed with cytokines IL-1β, IL-6, TNF and especially IL-8. Transduction with SIV-LMP1 resulted in a 50-fold induction of IL-12p70 secretion compared to transduction with SIV-GFP (from ~1 pg/ml to 50 pg/ml at 84 hours). Given the critical role of IL-12 in the stimulation of IFN-γ production, proliferation of T cells, and generation of cytotoxic T lymphocytes [23], using LMP1 as an adjuvant should result in increased DC activation and an enhanced T_H_1 immune response. This IL-12 induction is consistent with LMP1 inducing a constitutive CD40-like signal, a key role in Epstein Barr virus pathogenesis. Binding of CD40L to its receptor on immature DCs triggers DCs activation and maturation and increases DCs survival [17]. One of the cytokines upregulated in DCs activated by CD40L binding is IL-12, a cytokine responsible for polarizing CD4+ T cells to a T_H_1 phenotype [23]. Previous research with DNA vaccines showed that increasing the activation level of DC through CD40-CD40L interactions significantly enhances the intensity of cell mediated immunity and humoral immune responses [20,21,24-27]. Since IL-12 stimulates IFN-γ production, proliferation of T cells, and generation of cytotoxic T lymphocytes, it is logical that LMP1 and LMP1-CD40 result in increased DCs activation and a strong T_H_1 immune response.

The chemokines MIP-1α, MIP-1β, and RANTES play a critical role in innate immune control of HIV by DCs and macrophages [28,29]. Surprisingly, LMP1 and LMP1-CD40 were able to enhance these chemokines in the context of recombinant SIV virus infection. However, LMP1 was unique in its ability to induce IL-12p70, suggesting LMP1 would be a better inducer of T cell responses. Again, this chemokine secretion highlights the ability of LMP1 and LMP1-CD40 to increase the immune response during SIV infection of DCs and macrophages and suggests that these recombinant viruses may block viral replication while simultaneously enhances anti-HIV or SIV immune responses.

In addition to DC maturation and cytokine secretion, the immunogenicity of LMP1 and LMP1-CD40 was further confirmed by the coculture of virus infected DCs with autologous T cells for 12 days. This datum suggests that the LMP1 adjuvant gene cassette is able to convert a weakly immunogenic virus into a strongly immunogenic one that can augment T cell responses against viral antigens. By comparison, scSIV-LMP1-CD40 was less active in this assay, consistent with the overall weaker effect of scSIV-LMP1-CD40 in DCs compared to scSIV-LMP1 virus. This could reflect issues involved in the protein engineering of LMP1-CD40 that inhibit optimal CD40 signaling. Another explanation relates to the unique character of the LMP1 signaling domain. LMP1 signaling induces B cell stimulation without the requirement for costimulation, while CD40 signaling is costimulatory, needing additional signals for DC maturation and activation. As such, LMP1-CD40 may require additional stimuli, such as TLR agonists, a possibility that is currently being explored by our laboratory.

These data are encouraging, but it should be noted that LMP1 and the LMP1-CD40 chimera tend to induce qualitatively different responses on in terms of expression of surface markers and secretion of cytokines (Figures 3 and 4). Furthermore, the results obtained with DCs and macrophages do not always correlate directly (for example, compare CD40 and CD80 levels for Figures 3A to 3B). Despite these varied results, overall LMP1 and LMP1-CD40 show promise as SIV-based vaccine adjuvants able to enhance DC and macrophage immune responses.

While this approach is effective in inducing an immune response, there are also safety issues related to the use of LMP1. To improve the safety of this approach, a number of options are available to shut off LMP1 production in vivo. For example, the LMP1 and LMP1-CD40 systems could be regulated using an inducible promoter system [30,31]. Finally, the use of the nonhuman protein LMP1 as a molecular adjuvant may actually be advantageous compared to human-derived molecular adjuvants such as CD40, for example, by lowering the risk of autoimmune responses.

## Conclusions

Overall, these results provide the first evidence that LMP1 can act as a potent molecular adjuvant, providing a new class of adjuvant for use in recombinant vaccine strategies. In addition, LMP1 and LMP1 chimeras could be used as viral vector vaccine adjuvants or adjuvants for DNA or RNA based vaccines. Use of LMP1 for these or other subunit vaccine strategies is currently being explored. These vaccines could potentially target both DC and B cells, as B cell responses are also augmented by LMP1 expression, including the induction of T cell independent class switching [32].

## Methods

### Cells and media

Embryonic kidney (293T) cells were grown at 37°C under 5% CO2 in Dulbecco's modified Eagle medium (DMEM) supplemented with 10% fetal bovine serum (FBS), 2 mM L-glutamine, and antibiotics (100 U/ml penicillin and 100 μg/ml streptomycin), (referred to as complete medium). Human as well as rhesus macaque peripheral blood mononuclear cells (PBMCs) were prepared by Ficoll-Hypaque density centrifugation and maintained in RPMI medium (Hyclone, Logan, UT) supplemented with 5% human serum (Lonza, Allendale, NJ) and 10 mM HEPES (Invitrogen, Carlsbad, CA).

### Plasmid Construction

The construct SIV_mac_239 FS-ΔPR-ΔINEGFP (provided by Dr. David Evans) contains mutations in the gag-pol frameshift site (FS) and deletion in the protease (ΔPR) integrase (ΔIN) coding regions of the pol gene. The Nef coding region is replaced with GFP [33]. All constructs with the immunostimulatory genes LMP1 or LMP1-CD40 were cloned by overlap PCR and inserted into the SIV_mac_239 FS-ΔPR-ΔINEGFP vector using unique XbaI and SacII sites flanking the GFP gene. All viral clones were confirmed by DNA sequencing both before and after ligation into the viral vector. All DNA plasmids were purified with the Qiagen Endo-Free kit and checked for endotoxin levels prior to transfection.

### Preparation of viral stocks

Single-cycle virus stocks were prepared by harvesting the supernatant of 293T cells transfected with different viral plasmids. VSV-G trans-complemented single-cycle SIV was produced by co-transfection of 293T cells with the Gag-Pol expression construct pGPfusion, 5 μg of an expression construct for the Indiana or the New Jersey serotype of VSV-G and a full-length proviral DNA construct for each scSIV strain as previously described [33-35]. 293T cells were seeded at 5 × 10^6 ^cell per 100-mm dish in cell culture medium (Dulbecco's modified Eagle's medium [DMEM] supplemented with 10% fetal bovine serum [FBS], L-glutamine, penicillin and streptomycin) and transfected the following day with 5 μg of each plasmid using Genjet plus transfection Reagent (Signagen Laboratories, Iamsville, MD). Twenty-four hours after transfection, the plates were rinsed twice with serum-free medium and the cell culture medium was replaced with DMEM supplemented with 10% FBS. Twenty-four hours later, the cell culture supernatant was collected, clarified by centrifugation at 500 × *g *for 10 min, and filtered through a 0.45 μm-pore-size membrane (Millipore, Bedford, MA). To prepare high-titre stocks, viral particles were concentrated by repeated low speed centrifugation using YM-50 ultrafiltration units (Millipore, Bedford, MA). Aliquots (1 mL) of scSIV were cryopreserved at -80°C and the concentration of virus was determined by p27 antigen capture ELISA (Advanced BioScience Laboratories, Kensington, MD).

### Single-cycle SIV infectivity assays

One million CEM×174 cells were incubated with 100 ng p27 equivalents of scSIV in 100 μl volume for 2 hours at 37°C. Cultures were then expanded to a volume of 2 ml in R10 medium (RPMI supplemented with 10% FBS, L-glutamine, penicillin and streptomycin) and incubated in 24-well plates at 37°C for 4 days. Cells were treated with Fix and Perm reagents (BD Biosciences, San Jose, CA) and stained with FITC-conjugated SIV Gag-specific monoclonal antibody (Immunodiagnostics Inc. Woburn, MA). After staining, cells were fixed in 2% paraformaldehyde PBS and analyzed by flow cytometry to determine the frequency of SIV Gag-positive infected cells.

### Sodium dodecyl sulfate-polyacrylamide gel electrophoresis and Western blotting

Viral particle stocks were run on a 10% sodium dodecyl sulfate-polyacrylamide gel (Bio-Rad, Hercules, CA). Proteins were then transferred to nitrocellulose membranes (0.22 μm; GE Osmonics, Minnetonka, MN) and blocked (5% milk in PBS-0.2% Tween 20). The membranes were incubated individually with primary antibody overnight at 4°C. These antibodies included the following: (i) 1:100 dilution of mouse anti-EBV LMP1 monoclonal antibody (3H2104, a, b, c Santa Cruz Biotechnology, Santa Cruz, CA), (ii) 1:500 dilution of mouse anti-CD40 polyclonal antibody (C-20, Santa Cruz Biotechnology, Santa Cruz, CA), and (iii) 1:2,000 dilution of mouse anti-Gag p27 antibody, obtained through the National Institutes of Health AIDS Research and Reference Reagent Program (Germantown, MD) (SIVmac251 Gag monoclonal [KK64], catalogue no. 2321, from Karen Kent and Caroline Powell). Membranes were washed with PBS-0.2% Tween 20 and incubated with horseradish peroxidase (HRP)-conjugated goat anti-mouse antibody (Pierce, Rockford, IL) at a 1:5,000 dilution in blocking buffer. Following incubation in the secondary antibody, the membranes were washed and then incubated in HRP substrate (Pico chemiluminescence; Pierce). Membranes were placed on Whatman 3 MM filter paper and exposed to film (BioMax; Kodak, Rochester, NY).

### Preparation and transduction of monocyte-derived macrophages and dendritic cells

PBMCs from healthy blood donors (Continental Blood Services, Miami, FL) were isolated from buffy coats by density centrifugation using Ficoll-Hypaque (Amersham Pharmacia Biotech Inc., Piscataway, NJ). Cells were cultured at 2 × 10^6 ^cells/ml, in RPMI-1640 media supplemented with 10% decomplemented human AB serum (Biowhittaker, Walkersville, MD), 2 mmol/liter L-glutamine, 100 U/ml penicillin G and 100 μg/ml streptomycin (GIBCO BRL, Gaithersburg, MD), in a 5% CO_2 _atmosphere at 37°C. To isolate monocytes, PBMC underwent plastic adherence on T175 tissue flasks (Corning-Costar, Cambridge, MA). To generate enriched populations of monocyte-derived macrophages (macrophages) and monocyte-derived dendritic cells (DCs) the following procedures were performed. To generate macrophages, adherent cells were extensively washed and maintained for 24 h in medium supplemented with 10% heat-inactivated human serum. Adherent monocytes were washed, removed from the flask by gentle scraping, seeded onto 24-well plates at a density of 1 × 10^6 ^cells/well, and cultured for seven days. To generate immature DCs, plastic-adhered monocytes were cultured in GM-CSF, 800 U/ml and IL-4, 500 U/ml (R&D Systems, Inc., Minneapolis, MN) for 5 days, adding fresh GM-CSF and IL-4 on day 3. All cell culture reagents were endotoxin free.

### Virus transduction and flow cytometry

Immature DCs or macrophages were transduced at day 6. One million macrophages or DCs were incubated with 50 ng p27 equivalents of scSIV (MOI of 0.05) in 100 μl volume for 2 hours at 37°C. Cultures were then expanded to a volume of 2 ml in RPMI supplemented with 5% human serum, L-glutamine, penicillin and streptomycin and incubated at 37°C for 4 days. The culture supernatants of transduced macrophages or DCs were collected at various time points and stored at -80°C. Macrophages were stained on the plates, while DCs were harvested by gently resuspending the cells and staining with anti-CD40, anti-CD80, anti-CD83, anti-CD86, anti-CD11c or anti-HLA-DR or anti-CCR7 in fluorescence-activated cell sorter buffer (PBS supplemented with 3% fetal calf serum and 0.02% sodium azide). Intracellular staining for p27 was also performed to measure infectivity. Expression was monitored by flow cytometric analysis using a LSRII bioanalyzer (Becton Dickinson) and analyzed using the FlowJo software program (Tree Star, San Carlos, CA).

### Chemokine and cytokine assays

Cell culture supernatants were obtained from macrophages and DCs infected with different viruses at various time points. Supernatant samples were collected, centrifuged for 5 min at 13,000 × g to clarify, and the supernatant stored at -80°C. Concentrations of IL-1β, IL-6, IL-8, IL-10, IL-12p70 and TNF were measured using cytometric bead array (CBA) (BD Biosciences, San Jose, CA) according to the manufacturer's instructions.

### RT-PCR analysis of chemokine mRNA

For the measurement of MIP-1α (CCL3), MIP-1β (CCL4), and RANTES (CCL5) mRNA levels in the infected macrophages and DCs, quantitative RT-PCR was performed. Briefly, total RNA was prepared using the RNeasy kit (Qiagen Inc., Valencia, CA), and reverse transcribed in a 20 μl reaction containing 0.1 μg of total RNA, 0.1 μg of oligo(dT), 200 U of reverse transcriptase (Finnzymes, Finland) and 0.2 μM each of dATP, dCTP, dGTP and dTTP. After 1 hr incubation at 40°C, cDNA products were generated. Real-time PCR then was performed using the Power SYBR Green Supermix (Applied Biosystems) and the following primers: MIP-1α (CCL3)-specific primers, 5-GTC TGT GCT GAT CCC AGT GA-3 (forward) and 5-TTG TCA CCA GAC GCG GTG TG-3(reverse); MIP-1β (CCL4)-specific primers, 5-GTC TGT GCT GAT CCC AGT GA-3 (forward) and 5-GGA CAC TTA TCC TTT GGC TA-3 (reverse); RANTES (CCL5)-specific primers, 5-CCG CGG CAG CCC TCG CTG TCA TCC-3 (forward) and 5-CAT CTC CAA AGA GTT GAT GTA CTC C-3 (reverse). For normalization, GAPDH and β-actin real-time PCR was carried out on the same samples. Normalized mRNA levels for each transcript were calculated as (1/2ΔCt × 1,000), where ΔCt value = Ct (test mRNA) - Ct (GAPDH mRNA). To control for contamination with genomic DNA, parallel amplifications were performed in the absence of reverse transcriptase. These were uniformly negative.

### ELISPOT assay

IFN-γ ELISPOT assays were performed as previously described [36]. Briefly, isolated PBMCs were plated at a concentration of 100,000 cells per well in 96-well multiscreen plates (Millipore, Bedford, MA) that had been precoated with 0.5 g/ml of anti-IFN-γ monoclonal antibody (BD Biosciences, San Jose, CA). An SIV_mac_239 Gag peptide pool (15-mers overlapping by 11 aa (NIH AIDS Reagent Program)) was added at a final concentration of 5 μg/ml. Four wells containing PBMCs and complete medium alone were used as negative controls along with four positive controls with Phorbol Myristate Acetate (PMA, 5 ng/ml) and Ionomycin (500 ng/ml). Plates were incubated overnight at 37°C, 5% CO_2 _and developed as described previously (11). The numbers of spots per well were counted using an automated ELISPOT plate reader (CTL technologies), and the number of specific spot-forming cells (SFC), was calculated by subtracting the negative-control wells (mean plus 3 standard deviations). A value of 55 SFC/10^6 ^PBMC or greater (after subtraction of background) was considered positive.

### Statistics

Data were analyzed using PRISM 4.0 (GraphPad Software, La Jolla, CA) and expressed as the mean ± SEM. Statistical comparisons were analyzed by Student's t test. A p-value of 0.05 was chosen for statistical significance.

## Abbreviations

DC: dendritic cell; SIV: simian Immunodeficiency virus; LMP1: latent membrane protein 1; CBA: cytometric bead array

## Competing interests

The University of California San Diego has filed patent applications for the use of LMP1 and LMP1-CD40 as vaccine adjuvants, naming GWS and RSK as inventors.

## Authors' contributions

GWS, SG, and RSK designed the experiments and analyzed the data; SG, JT, LN, SK, and AR performed the experiments; SG, RSK, DE and GWS wrote the manuscript; and all of the authors read and approved the manuscript. RSK and GWS are listed as inventors on patent filings related to the use of LMP1 and LMP1-CD40 in vaccines.

## Supplementary Material

Additional file 1 **Calibrating infectivity and optimization of multiplicity of infection (MOI) of scSIV**. Fig. S1. To calculate the optimal infection dose, CEM cells were infected with a range of ng/million cells of VSV-G pseudotyped scSIV for 4 days and then stained with FITC anti-p27 antibody and analyzed by flowcytometry. Optimal infectivity was observed at 50 ng scSIV per million cells (MOI of 0.05). Click here for file

Additional file 2 **Table summarizing statistical analysis of cytokines secretion data**. Table S1. Statistical overview of cytokines secretion from a representative experiment of infected human DCs and macrophages (upper panel) and macaques DCs and macrophages (lower panel) with LMP1 and LMP1-CD40 adjuvanted virus. Human inflammatory cytokine quantitation was performed from the culture supernatants by cytometric bead array (CBA). P values are shown for any statistically significant differences between EGFP and LMP1/LMP1-CD40 viral constructs. Click here for file
