# Supplementary figures and images for: Latent Membrane Protein 1 as a molecular adjuvant for single-cycle lentiviral vaccines
Source: Retrovirology. 2011 May 18;8:39. doi: 10.1186/1742-4690-8-39 (PMC3118346; doi:10.1186/1742-4690-8-39)

## Slide 1
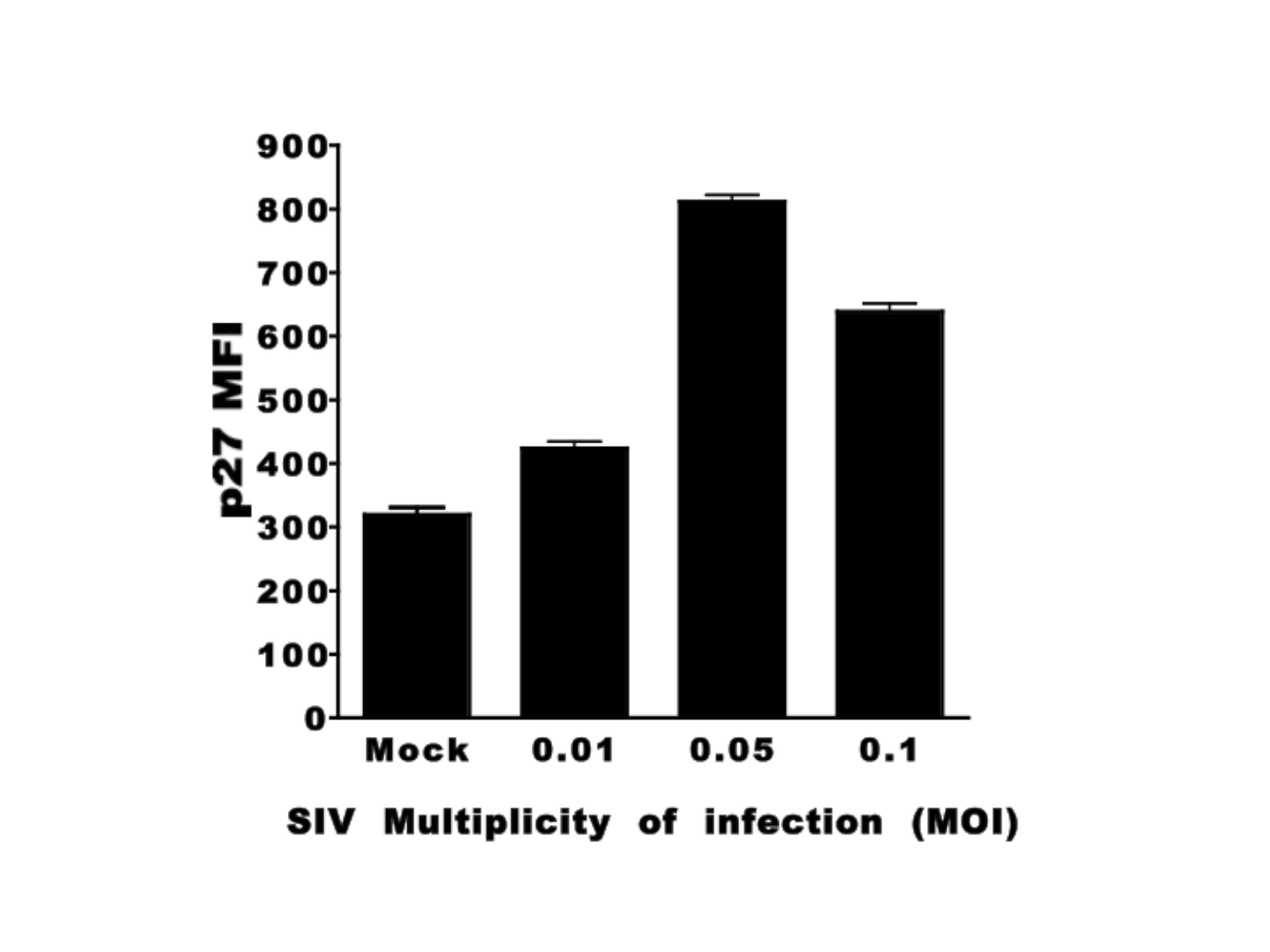

Supplement: Additional file 1 — Calibrating infectivity and optimization of multiplicity of infection (MOI) of scSIV. Fig. S1. To calculate the optimal infection dose, CEM cells were infected with a range of ng/million cells of VSV-G pseudotyped scSIV for 4 days and then stained with FITC anti-p27 antibody and analyzed by flowcytometry. Optimal infectivity was observed at 50 ng scSIV per million cells (MOI of 0.05). [file 1742-4690-8-39-S1.PPTX]

## Slide 1
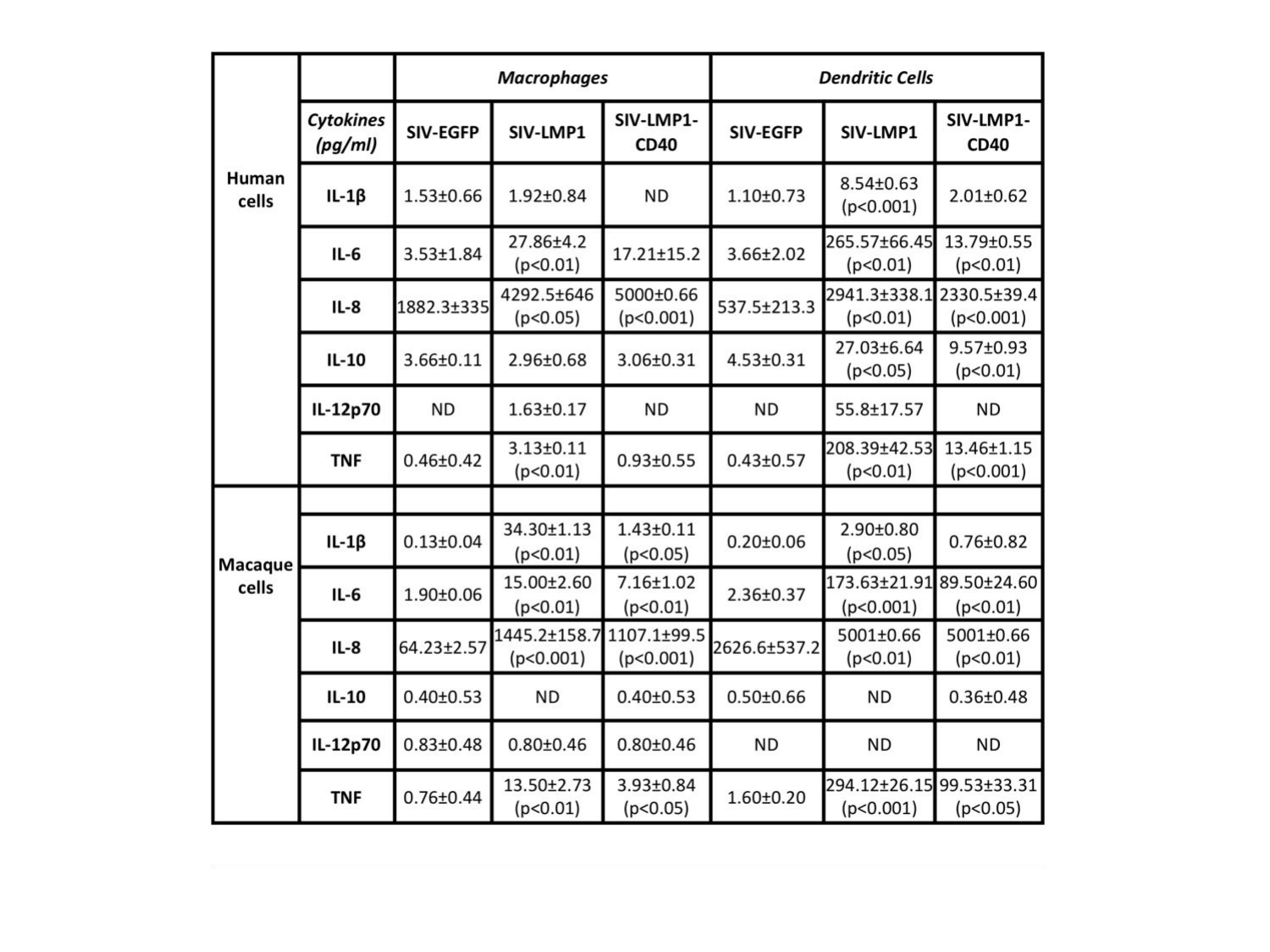

Supplement: Additional file 2 — Table summarizing statistical analysis of cytokines secretion data. Table S1. Statistical overview of cytokines secretion from a representative experiment of infected human DCs and macrophages (upper panel) and macaques DCs and macrophages (lower panel) with LMP1 and LMP1-CD40 adjuvanted virus. Human inflammatory cytokine quantitation was performed from the culture supernatants by cytometric bead array (CBA). P values are shown for any statistically significant differences between EGFP and LMP1/LMP1-CD40 viral constructs. [file 1742-4690-8-39-S2.PPTX]
